# Insulin-Like Growth Factor Binding Protein-5 in Physiology and Disease

**DOI:** 10.3389/fendo.2020.00100

**Published:** 2020-03-03

**Authors:** Cunming Duan, John B. Allard

**Affiliations:** Department of Molecular, Cellular, and Developmental Biology, University of Michigan, Ann Arbor, MI, United States

**Keywords:** IGF signaling, AKT, mTOR, PAPP-A, STC, IGF-dependent, IGF-independent action

## Abstract

Insulin-like growth factor (IGF) signaling is regulated by a conserved family of IGF binding proteins (IGFBPs) in vertebrates. Among the six distinct types of IGFBPs, IGFBP-5 is the most highly conserved across species and has the broadest range of biological activities. IGFBP-5 is expressed in diverse cell types, and its expression level is regulated by a variety of signaling pathways in different contexts. IGFBP-5 can exert a range of biological actions including prolonging the half-life of IGFs in the circulation, inhibition of IGF signaling by competing with the IGF-1 receptor for ligand binding, concentrating IGFs in certain cells and tissues, and potentiation of IGF signaling by delivery of IGFs to the IGF-1 receptor. IGFBP-5 also has IGF-independent activities and is even detected in the nucleus. Its broad biological activities make IGFBP-5 an excellent representative for understanding IGFBP functions. Despite its evolutionary conservation and numerous biological activities, knockout of IGFBP-5 in mice produced only a negligible phenotype. Recent research has begun to explain this paradox by demonstrating cell type-specific and physiological/pathological context-dependent roles for IGFBP-5. In this review, we survey and discuss what is currently known about IGFBP-5 in normal physiology and human disease. Based on recent *in vivo* genetic evidence, we suggest that IGFBP-5 is a multifunctional protein with the ability to act as a molecular switch to conditionally regulate IGF signaling.

## Introduction

The insulin-like growth factors (IGFs), including IGF-1 and IGF-2, are peptides that act throughout the vertebrate body via endocrine, paracrine, and autocrine signaling. IGFs bind to the IGF-1 receptor (IGF1R), a receptor tyrosine kinase that structurally resembles the insulin receptor (1). IGFs have very low affinity for the insulin receptor. The IGF signaling pathway regulates cell survival, differentiation, migration, and proliferation at the tissue level, and somatic growth, developmental progression, and aging at the organismal level (2–7).

A family of IGF-binding proteins (IGFBPs), regulates IGF bioavailability by binding to IGF ligands with equal or higher affinity than the IGF1R (8). Almost all of the IGFs in the extracellular environment, both in tissues and in the circulation, are found in complexes with IGFBPs (9, 10). There are six distinct types of IGFBPs in vertebrates, labeled IGFBP-1 through IGFBP-6. IGFBPs are found in all vertebrates studied to date, though not all species possess genes of all six IGFBP types, and some have multiple gene paralogs of some or all of the types (11). We have recently discussed the question of why the IGFBP family comprises such a large number of genes with substantial functional redundancy (11).

Here we review the current understanding of the structure, expression, regulation, and biological actions of IGFBP-5, which is the most highly conserved IGFBP family member. Like other IGFBP family members, IGFBP-5 binds to IGFs and can act to inhibit the interaction of the IGFs with the IGF1R and thereby reduce IGF signaling activity (11). While all IGFBPs have both shared and unique biological capabilities, spatiotemporal expression patterns, post-translational regulatory mechanisms, protein-protein interaction partners, etc., IGFBP-5 has one of the most diverse sets of biological actions of any IGFBP. This rich repertoire of activities makes IGFBP-5 an ideal representative of the IGFBP family by which to illustrate the range of mechanisms by which IGFBPs can modulate and fine-tune IGF signaling, and also carry out incidental functions that are independent of IGF binding. IGFBP-5 has been investigated for decades and discussed in a vast literature. We discuss evidence from a variety of vertebrate species, and in order to avoid confusion resulting from different gene/protein nomenclature systems, we will use the name “IGFBP-5” in all cases and explicitly indicate the species where necessary.

## Structure and Functional Motifs

**Figure 1:**
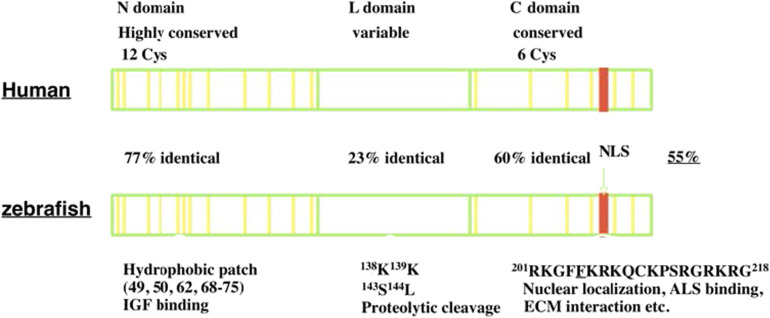
The structure of IGFBP-5. IGFBP-5 consists of 3 structurally domains: a highly conserved globular N-terminal domain, a central non-conserved linker domain, and a conserved C-terminal globular domain. The N-domain contains 12 conserved cysteine residues and a hydrophobic patch important for IGF binding. The L-domain contains several proteolytic cleavage sites. The C-domain contains six conserved cysteine residues, a RK-rich sequence (red) important for IGF binding, ALS binding, nuclear localization, and a thyroglobulin-like fold and other sites of interaction with ECM components.

IGFBP-5 was first identified and purified from human bone extracts and conditioned media collected from cultured human osteosarcoma cells (12, 13). It was subsequently cloned and characterized in a variety of vertebrate species (14–16). IGFBP-5 is found in all vertebrates studied to date, and its orthologs generally share the highest levels of amino acid sequence identity of any of the IGFBP types. Human IGFBP-5 contains 272 amino acids and most mammalian homologs of IGFBP-5 have either 272 or 271 amino acids. Human and zebrafish IGFBP-5 have an overall 55% sequence identity. Like all IGFBPs, mature human IGFBP-5 (252 amino acids) has a primary structure consisting of 3 domains, a highly conserved N-terminal domain, an unstructured linker (L-) domain that acts as a hinge, and a C-terminal structured domain that contains a thyroglobulin type-I repeat (11, 17) (Figure 1). The N- and C-terminal domains (N- and C- domains) are structurally stabilized by intradomain disulfide bonds between cysteine residues that are conserved across species, with 12 residues in the N- domain and 6 in the C domain (Figure 1). The L-domain is the least conserved region of the protein (17, 18).

Both the N- and C-domains in IGFBP-5 participate in IGF binding, but a fragment of the N-domain was found to bind IGF with substantial affinity, suggesting that it contains a sizable part of the interaction surface (19). The C-domain contains a highly conserved KR-rich sequence that overlaps with areas that contribute to IGF binding (20) (Figure 1). Binding of this region to heparin reduces the affinity for IGF by 17-fold, promoting release of bound IGF (21, 22). This region is also important for nuclear localization of IGFBP-5 (16, 23, 24) and ALS association (17, 25) (Figure 1). The C-domain also contains sites for binding various components of the extracellular matrix (ECM) (22). IGFBP-5 has been found to be localized within the ECM in tissues and has been shown to bind directly to a number of ECM proteins including types 3 and 4 collagen, Laminin, Fibronectin, Plasminogen Activator Inhibitor 1, Thrombospondin, and Osteopontin (26–28). The functional significance of ECM binding is discussed further below. The L-domain contains a number of proteolytic cleavage sites, phosphorylation, and O-glycosylation sites (17, 18, 29–31).

## Expression and Regulation

IGFBP-5 is expressed in a variety of different tissues throughout the body including lung, bone, muscle, testis, ovary, kidney, etc., with variations in different developmental stages and between species (18). IGFBP-5 expression is regulated by hormones in certain tissues and cell types, and is expressed constitutively in others. For instance, in mammary gland cells, IGFBP-5 expression is inhibited by prolactin (32). In osteoblast-like cells, parathyroid hormone upregulated IGFBP-5 expression (33). IGFBP-5 expression can also be upregulated by IGF signaling in vascular smooth muscle cells and other cell types (34, 35). In mouse mammary gland, IGFBP-5 expression is suppressed by the hormone prolactin and STAT-3 (32, 36, 37). IGFBP-5 mRNA has also been found to be regulated by several miRNAs (37–40).

Post-translational mechanisms also regulate IGFBP-5 in important ways (18). IGFBP-5 was shown to be phosphorylated on several serine residues *in vivo*, which reduced its binding affinity for heparin but not for IGFs (41). In the extracellular environment, a number of specific proteases cleave IGFBP-5. In some cases, proteolysis of IGFBP-5 is inhibited by IGF binding (42). The zinc-dependent metalloproteinases pregnancy-associated plasma protein-a (PAPP-A) and PAPP-A2 have been shown to cleave IGFBP-5 at a single site in the L-domain (31, 43, 44). Unlike IGFBP-4, which is only susceptible to cleavage by PAPP-A when it is bound to IGF, IGFBP-5 is cleaved by both proteases regardless of IGF binding (31, 43). PAPP-A2 knockout mice had 2-fold higher levels of IGFBP-5. Interestingly, these mice also exhibited a 15-fold reduction in IGFBP-3 levels and a 60% increase in total IGF levels (45). The deletion of PAPP-A2 in osteoblast cells in mice led to a significant reduction in growth as measured by both body mass and tail length (46). The proteolytic regulation of IGFBP-5 by PAPP-A and PAPP-A2 is conserved in zebrafish (47). In addition to PAPP-A and PAPP-A2, a number of other proteases have been reported to degrade IGFBP-5. These include thrombin, elastase, cathepsin G, C1s, ADAM 9, ADAM 12s, MMP-1, and MMP-2 etc. (48–53).

Interactions with extracellular matrix (ECM) and cell surface proteins are also important for IGFBP-5 activity. A number of studies have demonstrated a link between IGFBP-5 ECM binding and its enhanced potentiation of IGF signaling. IGFBP-5 associated with the cell culture substratum of fibroblasts was found to potentiate the cellular growth promoting effects of IGF signaling (26). Binding to the ECM component vitronectin enhanced IGFBP-5's potentiation of IGF signaling in smooth muscle cells, and a mutant form of IGFBP-5 that did not bind to vitronectin did not produce this effect (54). IGFBP-5 mutants with reduced ECM binding ability had a reduced ability to potentiate IGF signaling *in vitro* (30, 55). Some ECM components also influence the biosynthesis of IGFBP-5. The ECM component fibronectin in the culture substrate was found to upregulate expression and secretion of IGFBP-5 in porcine smooth muscle cells (56).

Early *in vitro* studies showed that its binding to heparin-like glycosaminoglycans protected IGFBP-5 from proteolytic degradation in media conditioned by human dermal fibroblasts (57). This mechanism may allow IGF/IGFBP-5 complexes bound to proteoglycans in the ECM to avoid proteolysis for an extended period. It was suggested that IGFBP-5 may serve as a reservoir of IGF in tissues for later release when needed (30, 58–60). This may be important in bone tissue, where the IGF-IGFBP-5 complex is found in large quantities, binding to hydroxyapatite (58–60). A recent study found that IGF-1 released from the bone matrix promotes the differentiation of mesenchymal stem cells into osteoblasts, aiding bone formation during bone remodeling (61).

## Endocrine Role of IGFBP-5

**Figure 2:**
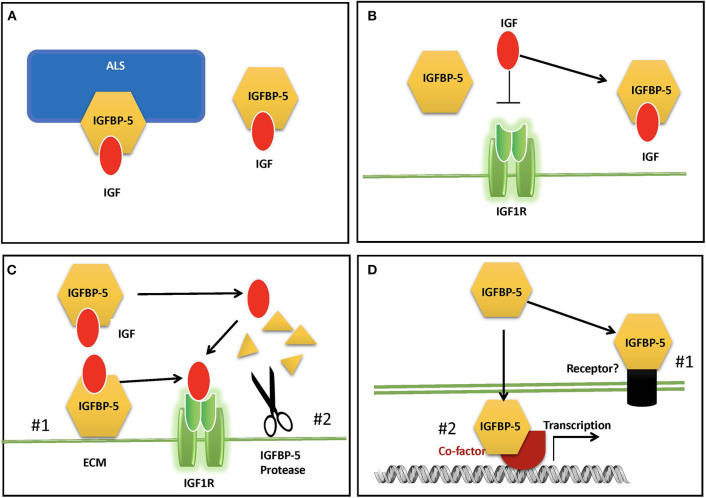
Proposed modes of IGFBP-5 actions. **(A)** IGFBP-5 modulates circulating IGFs by forming a binary complex with IGF or a ternary complex with IGF and acid labile subunit (ALS) in the blood. **(B)** IGFBP-5 inhibits IGF signaling in target cells by sequestrating IGF away from the IGF1R. **(C)** IGFBP-5 potentiates IGF signaling in target cells by #1) releasing of bound IGF to the IGF-1R upon interaction with ECM and cell surface molecules or #2) protease-mediated IGFBP-5 proteolysis. **(D)** IGF-independent action of IGFBP-5 via #1) its putative membrane receptor(s) or #2) interacting with co-factors in the nucleus.

In adult human blood, IGFs are found at mean concentrations that are around 1,000-fold higher than insulin (9). Therefore, despite the low cross-reactivity of IGFs with the insulin receptor, if all circulating IGFs were free to interact, the hypoglycemic effects would overwhelm the effects of insulin itself. In addition, free IGF has a half-life in circulation of around 10 min (4, 62). Around 1% or less of circulating IGFs are free, and the remaining >99% are complexed with one of the IGFBPs (9). Binary complexes of IGF and IGFBP extend the half-life of the IGF to roughly 30 min, but they also facilitate the departure the IGF to be delivered to tissues (4, 62). Like IGFBP-3, which is the dominant IGFBP in the circulation, IGFBP-5 can bind to IGF alone or in a ternary complex with IGF and an 85 kDa glycoprotein called Acid Labile Subunit (ALS) (25). IGF within the ternary complex has a greatly prolonged half-life, and the complex is too large (around 150 kDa) to exit from the circulation, and thus it is able to maintain a circulating reservoir of IGF (63) (Figure 2A). About 75–80% of circulating IGF is found in a ternary complex with IGFBP-3 or−5 (9). When both IGFBP-3 and IGFBP-5 were knocked out in mice (64), or when ALS itself was deleted (65), the ternary complex was absent, and serum IGF levels were greatly reduced. However, there was only a modest reduction in growth, due to compensatory mechanisms by other IGFBPs.

Global overexpression of IGFBP-5 in mice, which resulted in a ~4-fold increase in circulating IGFBP-5, caused a severe body growth reduction both prenatally and postnatally, significant neonatal mortality, reduction of fertility in females, and a 30% reduction in skeletal muscle weight (66). This phenotype was consistent with the notion that IGFBP-5 inhibits IGF signaling by sequestering IGF away from the IGF1R (4, 10). However, the relevance of this overexpression phenotype to the physiological role(s) of endogenous IGFBP-5 is unclear. Knockout of IGFBP-5 in mice did not result in altered body growth compared with wildtype, and it was assumed that other IGFBPs compensate for the lack of IGFBP-5 (67). This is supported by that fact that mice lacking IGFBP-3,−4, and−5 had somewhat reduced growth, changes in metabolism, a significant reduction in circulating and bioactive IGF-1 levels, which may explain the reduced body growth (64). Another possible explanation is that altered physiological and/or pathological states resulting from global dysregulation of IGF signaling led to growth inhibition.

## Roles of Local Igfbp-5 In Regulating Igf Actions

Locally expressed IGFBP-5 can inhibit or enhance IGF biological activity by modulating their interaction with the IGF-I receptor (Figures 2B,C). IGFBP-5 is the most abundant IGFBP in bone tissues (68) and there is a host of *in vitro* findings in the literature regarding IGFBP-5 actions in osteosarcoma cells. When added in combination with IGF-I to cultured human osteosarcoma cells, IGFBP-5 was found to inhibit IGF-I-induced cell growth [(69); 29]. Likewise, stable overexpression of IGFBP-5 was found to inhibit mouse osteosarcoma cell proliferation (70). In mesenchymal stem cell cultures, exogenously added IGFBP-5 and endogenously overexpressed IGFBP-5 inhibited osteoblast differentiation, while an IGF-binding deficient IGFBP-5 mutant did not have this effect (71). When IGFBP-5 was overexpressed in transgenic mice under the control of a bone specific osteocalcin promotor, osteoblast function was impaired, leading to reduced trabecular bone volume and reduced mineral densities (72). On the other hand, IGFBP-5 was also found to potentiate IGF-I-induced DNA synthesis and differentiation in bone cells (12, 13, 73, 74). The potentiating effects of IGFBP-5 were attributed to its ability to bind to the bone extracellular matrix (ECM) since IGFBP-5 has a high affinity for hydroxyapatite (73, 75). Given that IGFBP-5 is already abundantly expressed in bone cells, interpretations of data from the addition of exogenous IGFBP-5 or overexpression of IGFBP-5 are not always straightforward. Indeed, IGFBP-5 knockout mice had minimal changes in bone (64). Another complication is the presence of one or more IGFBP-5 protease(s) secreted by these cells and the fact that some IGFBP-5 fragments can exert IGF-independent actions in bone cells [(44, 76, 77), see below]. Moreover, different IGFBP-5 fragments might have different activities in osteosarcoma cells: while the N-terminal domain fragment inhibited cell proliferation and induced apoptosis, its C-terminal domain inhibited cell migration and metastases (78).

**Figure 3:**
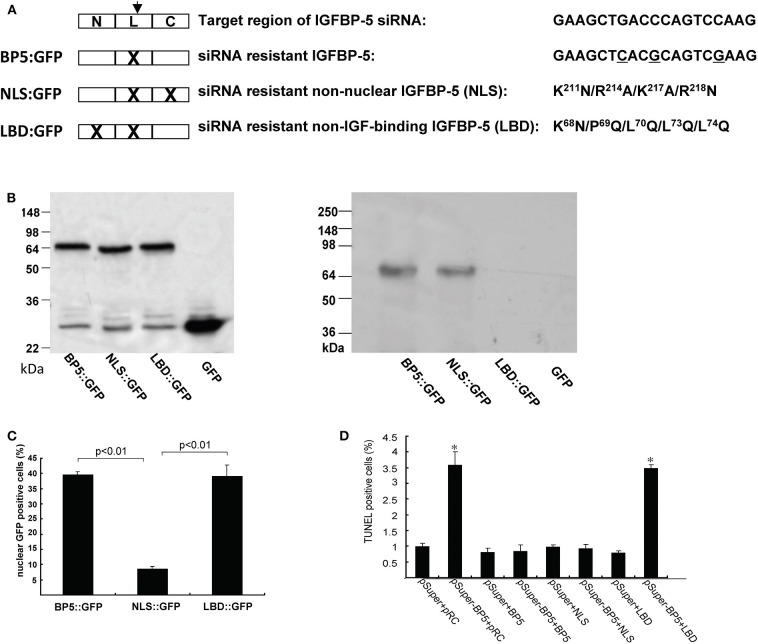
IGFBP-5 regulates osteosarcoma cell survival by binding to IGFs. **(A)** Schematic diagram showing the structure of IGFBP-5 and three siRNA resistant IGFBP-5 expression constructs. Three point mutations were introduced into the target region of the siRNA to make the IGFBP-5 resistant to the RNA interference (BP5::GFP). Since the mutations are on the third position of the codons, the amino acid sequence is unchanged. Nuclear localization mutations (NLS) and IGF ligand binding deficient mutations (LBD) were further introduced into the siRNA resistant BP5::GFP plasmid, resulting in the NLS::GFP and LBD::GFP construct. **(B)** Left panel: Western immunoblot showing the expression levels of the three siRNA resistant IGFBP-5::GFP proteins in transfected U2 osteosarcoma cells. Right panel: Western ligand blot using DIG labeled IGF-I showing the ligand binding capability of BP5::GFP, NLS::GFP and LBD::GFP. Note the lack of IGF binding of LBD::GFP. pRC is the empty GFP vector. **(C)** Percentage of transfected cells with nuclear GFP signal. **(D)** The three siRNA resistant constructs were co-transfected into human U2 osteosarcoma cells with pSuper (the empty siRNA vector) or pSuper-BP5 (IGFBP-5 siRNA plasmid). The percentages of TUNEL positive cells were quantified. ^*^p < *0.05* compared with the pSuper control group.

Yin et al. (79) investigated the role of endogenous IGFBP-5 using a siRNA based gene knockdown approach. They found that knockdown of IGFBP-5 increased osteosarcoma cell apoptosis. To further elucidate the mechanism underlying this action of IGFBP-5, we recently generated an expression plasmid encoding a siRNA resistant form of human IGFBP-5 (BP-5::GFP) (Figure 3A). The introduction of the siRNA-resistant IGFBP-5 into IGFBP-5 knocked down cells rescued cells from apoptosis (Figures 3B,D), suggesting that IGFBP-5 is both required and sufficient for maintaining osteosarcoma cell survival. IGFBP-5 is not only secreted but also localized in the nuclei and the IGFBP-5 has nuclear activity (see below). To determine the mechanism underlying IGFBP-5 actions, a ligand-binding deficient (LBD) and a nuclear localization deficient (NLS) form of IGFBP-5 (i.e., LBD::GFP and NLS::GFP) were engineered in the BP-5::GFP plasmid background (Figure 3A). The NLS mutant bound normally to IGF-1 but had greatly reduced nuclear localization. The LDB mutant failed to interact with IGF-I, but showed similar nuclear localization (Figures 3B,C). The introduction of NLS::GFP but not LBD::GFP into IGFBP-5 knocked down human osteosarcoma cells rescued them from apoptosis (Figure 3D). These results suggest that endogenous IGFBP-5 regulates osteosarcoma cell survival by binding to IGFs and enhancing IGF action.

Involution of the mammary gland is the process by which a burst of apoptosis of mammary epithelial cells accompanied by ECM remodeling returns the gland to its condition before pregnancy (80). IGFBP-5 is upregulated in mammary epithelial cells during involution, where it may inhibit IGF signaling (32, 81, 82). This reduction of IGF signaling removes a key survival signal and thereby promotes mammary cell apoptosis (81). Indeed, IGFBP-5 knockout mice exhibited delayed mammary gland involution, as well as an enhancement in alveolar bud formation when ovariectomized mice were regularly injected with ovarian hormones to strongly promote mammary development (67). Transgenic overexpression of IGFs in mammary tissue led to a delay in involution (83, 84). Overexpression of IGFBP-5 in mammary gland resulted in a 50% reduction in mammary cell number and milk production, along with a reduction in the activation of downstream IGF signaling, an increase in expression of the proapoptotic caspase-3 and a decrease in expression of antiapoptotic components (85). These findings suggest that IGF-1 promotes alveolar bud formation in normal pubertal mammary gland development and inhibits mammary cell apoptosis, and that IGFBP-5 inhibits these IGF actions (67). The upregulation of IGFBP-5 has been found to promote apoptosis in other tissues as well, including neurons and cardiomyocytes (86, 87).

Genetic studies in zebrafish have shown that IGFBP-5 potentiates IGF signaling in epithelial cells *in vivo* (88). One of the two zebrafish IGFBP-5 paralogous genes, IGFBP-5a is specifically expressed in a population of epithelial cells, known as Ca^2+^ transporting ionocytes or NaR cells, whose role is to take up Ca^2+^ from the aquatic environment to maintain body Ca^2+^ homeostasis (89–91). When environmental [Ca^2+^] becomes scarce, these normally non-dividing and quiescent ionocytes reenter the cell cycle and begin to proliferate, producing a much larger capacity for Ca^2+^ uptake and allowing the embryos/larvae to survive under these stressful conditions (90, 92). This proliferative response is mediated by IGF signaling which is activated exclusively in these cells in response to low Ca^2+^ stress (90, 92). Genetic deletion of IGFBP-5a prevented the activation of IGF signaling in ionocytes under low [Ca^2+^] stress (88). This prevented the adaptive proliferation of ionocytes, and the IGFBP-5 null embryos were therefore unable to survive under low [Ca^2+^] stress (88). Reintroduction of wild-type zebrafish IGFBP5a in the mutant cells restores their adaptive proliferation. However, a ligand binding deficient IGFBP5a mutant had no such effect, suggesting that locally expressed IGFBP-5a regulates epithelial cell proliferation by binding to the IGF ligand and promoting IGF signaling under low [Ca^2+^] stress. This action appears to be conserved in human cells because knockdown of IGFBP-5 expression in human colon carcinoma cells reduced their proliferative response to IGF-2 stimulation (88). *In vivo*, expression of human IGFBP-5 in mutant zebrafish increased ionocyte proliferation, whereas two cancer-associated human IGFBP-5 mutations with impaired IGF binding ability (93) had no effect (88). This type of local regulation of IGF signaling by IGFBP-5 under certain stressful and/or pathophysiological states may be a common mechanism. It was reported that castration of male mice induces local IGFBP-5 expression in prostate tissue and the elevated IGFBP5 increases IGF action and promotes prostate cancer progression (94, 95). Likewise, an increase in local IGFBP-5 expression has been shown in resection-induced adaptive colon growth (96).

Another example of the IGF potentiating effects of IGFBP-5 is seen in muscle development, where IGF-2 has been found to promote proliferation of myoblast cells as well as their differentiation into mature muscle cells (97). Myoblasts secrete IGF-2 during differentiation, which acts in an autocrine fashion (98). Upregulation of IGFBP-5 preceded upregulation of IGF-2 in these cells and knockdown of IGFBP-5 blocked myogenic differentiation, suggesting that IGFBP-5 was necessary to guide the activity of IGF signaling toward differentiation (99). This action of IGFBP-5 required its ability to bind to IGF-2 because an IGF-binding deficient form of IGFBP-5 had no such effect (99). IGFBP-5 was found incorporated into the ECM in cultures of fetal fibroblasts, and ECM binding facilitated its potentiation of the growth promoting effects of IGF on these cells (26). In porcine vascular smooth muscle cells, IGFBP-5 potentiated the positive effect of IGF signaling on DNA synthesis, whereas IGFBP-4 had an inhibitory effect on IGF action (35). In a mouse model of prostate cancer, upregulation of IGFBP-5 following androgen withdrawal by castration was found to potentiate IGF signaling *in vivo*, which led to faster progression to androgen dependence (95).

## Emerging Role of IGFBP-5 As a Molecular Switch That Turns on or Off IGF Signaling

**Figure 4:**
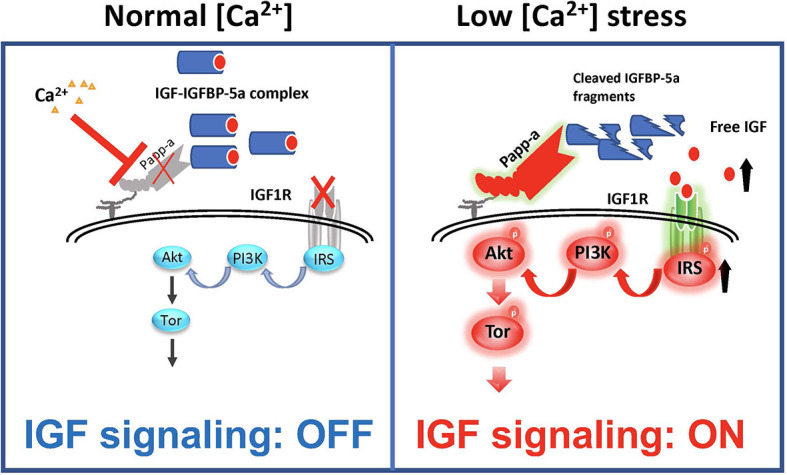
IGFBP-5 is part of a molecular switch that turns IGF signaling on or off in target cells. Zebrafish IGFBP-5a and the conserved zinc metalloproteinase Papp-aa are expressed in ionocytes. Left panel: under normal [Ca^2+^] conditions, Papp-a proteolysis activity is low. Igfbp5a is intact and it inhibits IGF signaling in these cells by binding to IGFs and prevents their binding to the IGF1R in ionocytes. Right panel: under low [Ca^2+^] conditions, Papp-a activity is increased. This increases IGFBP-5a proteolytic cleavage and releases IGFs from the IGFBP-5a/IGF complex to activate IGF-1 receptor-mediated PI3 kinase-Akt-Tor signaling and promotes ionocytes to proliferate.

As discussed above, IGFBP-5 has been shown to be able to inhibit and potentiate IGF signaling in different cell types and/or contexts. When IGFBP-5 was overexpressed *in vivo* in mice, opposite effects were seen on bone formation rate in the periosteum and endosteum suggesting opposite effects on osteoblast proliferation or survival in these regions (100). In vascular smooth muscle cells, IGFBP-5 inhibited IGF-1-dependent DNA synthesis, while it potentiated IGF-1-dependent cell migration (101). How can these seemingly opposite effects be explained? A recent study by Liu et al. (47) has elucidated that while IGFBP-5 inhibits IGF signaling in zebrafish Ca^2+^ transporting ionocytes under normal conditions, it potentiates IGF signaling under low [Ca^2+^] stress. In addition to IGFBP-5a, these ionocytes highly express Papp-aa, a zebrafish homolog of the IGFBP protease PAPP-A. Treatment of fish with ZnCl_2_ or batimastat, two metalloproteinase inhibitors (102), inhibited the low [Ca^2+^] stress-induced ionocyte proliferation, suggesting that Papp-aa protease activity is critical. Genetic deletion of Papp-aa abolished low [Ca^2+^]-induced ionocyte proliferation, while it had little effect on ionocyte proliferation when fish were kept in normal conditions (47). Loss of Papp-aa expression or activity resulted in diminished IGF1 receptor-mediated Akt-Tor signaling in ionocytes in response to low [Ca^2+^] stress (47). This phenotype was similar to the igfbp5a^−/−^ mutant fish (88). Biochemically, Papp-aa cleaved IGFBP-5a. Re-introduction of wild-type Papp-aa rescued cell proliferation and IGF signaling, while a protease deficient Papp-aa mutant could not rescue the ionocyte proliferative response (47). Because igfbp5a mRNA levels in each ionocyte did not change under different [Ca^2+^], Liu et al. (47) speculated that a [Ca^2+^]-dependent post-transcriptional regulatory mechanisms must block Papp-aa proteolytic activity when [Ca^2+^] is sufficient (Figure 4). This idea was supported by the fact that treatment of fish with NBI-31772, an aptamer that can displace and release IGF from the IGF/IGFBP complex (103) promoted ionocyte proliferation under normal [Ca^2+^]. NBI-31772 treatment significantly also increased the levels of phospho-Akt and phospho-pS6 activity in ionocytes (47). These data suggest that latent IGF is present and that the limiting step under normal [Ca^2+^] is the release of bioavailable IGFs.

Bony fish produce a hormone called stanniocalcin (STC) in response to high serum [Ca^2+^] and it inhibits Ca^2+^ uptake (104). The mammalian STC homologs STC-1 and STC-2 were found to strongly inhibit PAPP-A proteolytic activity (105, 106). In zebrafish, the levels of STC-1 mRNA are regulated by Ca^2+^ levels (107). Although it remains to be determined whether endogenous zebrafish STC1 regulates Papp-aa activity in ionocytes, over expression of STC-1 and STC-2 in ionocytes in zebrafish inhibited Papp-aa-dependent activation of ionocyte proliferation (47). Based on these findings, it was postulated that Papp-aa-mediated IGFBP-5a proteolysis functions as a [Ca^2+^]-regulated molecular switch to conditionally activate IGF signaling in ionocytes (Figure 4). Under normal [Ca^2+^] conditions, Papp-a proteolysis activity is inhibited and Igfbp5a is mostly intact. The intact IGFBP-5a inhibits IGF action by binding to IGFs and preventing their binding to the IGF1 receptor. Under low [Ca^2+^] conditions, however, Papp-a activity is increased, possibly due to changes in STC1 levels. This increases IGFBP-5a proteolytic cleavage and releases IGFs from the Igfbp5a/IGF complex. This in turn activates IGF-1 receptor-mediated PI3 kinase-Akt-Tor signaling and promotes ionocyte proliferation (47).

## IGF-Independent Actions of IGFBP-5

A number of reports have suggested that IGFBP-5 can act via IGF-independent mechanisms (Figure 2D). As discussed above, overexpression of IGFBP-5 in mice resulted in significant prenatal and postnatal whole body growth inhibition, which is consistent with the idea that IGFBPs inhibit IGF signaling by inhibiting IGF-IGF1R binding (66). However, overexpression of an IGFBP-5 mutant that lacks binding affinity for IGF also produced significant inhibition of growth, despite the lack of any effects on the IGF signaling pathway (108). This supports the notion that that IGFBP-5 can inhibit growth via an IGF-independent mechanism. Based on *in vitro* studies, IGFBP-5 has been suggested as a bone growth factor and exerts biological activities that are independent of IGFs (109). A number of early reports have suggested that IGFBP-5 binds to its own cell surface receptor, and indeed, IGFBP-3, the most closely related paralog of IGFBP-5, has been found to interact functionally with the type V transforming growth factor beta receptor (LRP-1), which may mediate the IGF-independent growth inhibitory effect (110–112). IGF signaling is crucial for skeletal growth, and both IGF-1 and IGFBPs, including IGFBP-5 are expressed in bone tissue. Binding of IGFBP-5 to sites on the bone cell surface was found to increase proliferation even in the presence of IGF analogs that have 100-fold reduced binding affinity for IGFBPs, suggesting that IGF binding was not required for this action (73). It was suggested that IGFBP-5 may bind to specific receptors on the surface of osteoblastic cells (113). However, to date, no specific IGFBP-5 cell surface receptor has been identified molecularly. But functional interactions with some cell surface proteins have been reported. IGFBP-5 interacted directly with alpha2beta1 integrin on human breast cancer cells *in vitro* and promoted survival and adhesion but inhibited migration (114). Some IGFBP-5 proteolytic fragments were reported to exert IGF-independent actions in cultured bone cells [(76, 77); 44]. In vascular smooth muscle cells, IGFBP-5 was shown to promote cell migration by an IGF-independent mechanism that was facilitated by binding to cell surface proteoglycans (101).

IGFBP-5 contains a conserved nuclear localization sequence (NLS) motif (115), and was shown to be imported in to the nucleus *in vitro* via an importin beta-dependent pathway (23). In addition to its functional NLS, IGFBP-5 was also found to possess transactivation activity in cell culture experiments (24, 116). IGFBP transactivation and nuclear localization are also found across species ranging from zebrafish to the cephalochordate amphioxus (117). Regulation of target genes by nuclear IGFBP-5 *in vivo*, and possible physiological roles of this activity have yet to be elucidated, but the conservation of IGFBP nuclear transactivation activity across chordate evolutionary history lends credence to the idea that such physiological roles may exist. A yeast two hybrid screen for nuclear protein-protein interaction partners found that IGFBP-5 interacts with nuclear protein FHL2 *in vitro*, but the physiological relevance of this interaction is unclear (118). IGFBP-5 was also found to interact in the nuclei of osteoblast-like cells *in vitro* with the vitamin D receptor (119). This interaction reduced the cellular response to 1,25-dihydroxyvitamin D3 which normally promotes cell cycle exit, differentiation, and expression of bone matrix proteins in these cells, and this effect was seen only when the cells produced IGFBP-5 endogenously and not when it was added exogenously (119).

## IGFBP-5 in Pathology and Disease States

IGFBP-5 has been found to be altered in various disease states (120–123), providing the possibility of using this protein as a marker of disease progression, and hinting that altered IGFBP-5 expression may have pathophysiological relevance. Altered levels of IGFBP-5 have been detected in many types of cancer. Ding et al. (93) have identified over 20 non-synonymous IGFBP-5 mutations in a variety of cancer cell lines. These include frame-shift and non-sense mutations. Several of them, including G223R and W242^*^ were speculated to have lost IGF binding ability. IGFBP-5 has been found to indicate a poor prognosis in patients with several types of cancer (124). IGFBP-5 levels are significantly elevated in osteosarcoma cells that exhibit high metastatic potential (125, 126). However, others found that IGFBP-5 expression inhibited osteosarcoma tumor growth and metastasis (78, 127). In gastric cancer, upregulation of IGFBP-5 was found to partially mediate the action of the PBX/Knotted Homeobox 2 tumor suppressor (128). In papillary thyroid carcinoma, IGFBP-5 was reported to promote cell growth, and miR-204-5p, which inhibits growth by suppressing IGFBP-5, was downregulated in these cells (39). In MCF-7 breast cancer cells, IGFBP-5 promoted cell survival and adhesion via an IGF-independent mechanism (114). A genome wide association study found an SNP allele associated with reduced IGFBP-5 expression and this SNP conferred increased susceptibility to breast cancer, which is consistent with the role of IGFBP-5 in mammary gland discussed above (129). IGFBP-5 has been found to both inhibit and promote cancer cell growth *in vitro* (130–135). It is possible that the expression of IGFBP-5 protease(s) may be important for determining the context-specific effects of IGFBP-5.

IGFBP-5 may play a role in the pathogenesis of atherosclerosis, which is a process of inflammatory tissue remodeling within the matrix of the arterial wall that is the top cause of cardiovascular disease and aging-related mortality in humans (136). A cross-sectional case-control study found a positive association between circulating IGFBP-5 levels and coronary heart disease (137). Overexpression of PAPP-A, whose only known substrates are IGFBP-2, -4, and -5, in the arterial smooth muscle of mice enhanced the progression of atherosclerotic lesion development (138). PAPP-A knockout mice are protected from atherosclerosis as well (139). Conflicting results have been found in mouse models in which other components of the IGF system have been manipulated, and there are indications that circulating IGF may be protective rather than pro-atherosclerotic (140). Local IGF signaling plays an important role in atherosclerosis by stimulating the proliferation of vascular smooth muscle cells and their migration into the arterial intima where they contribute to the formation of atherosclerotic plaques (141–143). Local IGF signaling in the arterial wall and in atherosclerotic plaques is regulated by multiple IGFBPs including IGFBP-5 (101, 144). IGFBP-2 and -4 inhibit IGF signaling in VSMCs but IGFBP-5 enhances it (35, 101). IGFBP-5 expression is upregulated in atherosclerotic plaques and IGFBP-5 protein is found in large quantities associated with ECM within atherosclerotic plaques (56, 145). IGFBP-5 is known to bind to ECM components PAI-1 and osteopontin, which have both been found in atherosclerotic plaques and have been shown to promote atherosclerosis in loss of function studies (28, 146, 147). ECM associated IGFBP-5 potentiates IGF signaling, and IGF signaling can upregulate expression of IGFBP-5, so it is possible that a positive feedback loop could contribute to atherogenesis (28, 143).

IGFBP-5 was shown to be upregulated in lung tissue from patients with idiopathic pulmonary fibrosis (IPF), and exogenous IGFBP-5 also stimulates the secretion of ECM components by IPF lung fibroblasts (148). This effect was independent of IGF-binding but also did not require translocation into the nucleus (149). Exogenous and endogenously expressed IGFBP-5 was found to increase the expression of ECM component genes and pro-fibrotic genes in primary human IPF fibroblasts *in vitro* (150). IGFBP-5 was also shown to increase expression of its own gene in these cells, leading to a positive feedback loop that may play a role in IPF pathogenesis (150).

The role of IGFBP-5 in both atherosclerosis and fibrosis may be linked to the induction of cellular senescence (145, 151). Aged artery walls are more susceptible to atherosclerosis and hypertension which may be related to accumulation of senescent cells and the resultant compositional changes in the subendothelial matrix (136). The accumulation of senescent cells in the arteries of children with the premature aging disease Hutchinson-Gilford Progeria seems to be the cause of their severe accelerated atherosclerosis and premature death from resulting stroke or heart attack before age 20 (152, 153). Senescent cells exhibit a senescence-associated secretory phenotype that is characterized by excessive production of ECM components, and this may play a role in tissue fibrosis (122). IGFBP-5 was upregulated in senescent human umbilical vein endothelial cells and knock down of IGFBP-5 partially reversed the senescence, suggesting a role for IGFBP-5 in promoting cellular senescence (145). The accumulation of senescent cells may play a causal role in many aspects of the vertebrate aging process, which is known to be promoted by IGF signaling (136). Future research will determine the extent to which IGFBP-5 may be involved in linking IGF signaling to aging-related changes in tissues and the pathology of aging related diseases.

## Conclusions and Prospects

IGFBP-5 is a multifunctional protein that is capable of regulating IGF signaling both positively and negatively in different tissues and cells. It can also promote, or inhibit cell survival, proliferation, migration, etc. via mechanisms independent of IGF binding. The range of reported IGFBP-5 actions in different cell types can be daunting to understand. There are several possible explanations for the plethora of IGFBP-5 activities: (1) many actions of IGFBP-5 have been reported only in immortalized cell lines *in vitro*, and as such, they can only be accepted as potential actions with uncertain physiological relevance until they are confirmed *in vivo*; (2) different study methodologies may demonstrate opposite findings as result of a downstream effects depending sensitively on the dose of IGFBP-5, i.e., a small amount IGFBP-5 may potentiate IGF signaling while a large enough excess of IGFBP-5 may switch to inhibition; (3) in some cases, exogenous and endogenous IGFBP-5 may act through different mechanisms, possibly as a result of different posttranslational modifications, etc. (79); and (4) IGFBP-5 can act as the pivot point in a switch between regulated states of inhibition and activation of downstream signaling (Figure 4). For instance, IGFBP-5a inhibits IGF signaling in zebrafish ionocytes under normal physiological medium, while it potentiates IGF signaling in the same cells when it is proteolytically cleaved by Papp-aa under low [Ca^2+^] stress (47).

We will not understand why IGFBP-5 has IGF-independent actions until these actions are fully elucidated *in vivo*. However, it is worth considering that some of these actions may have arisen as a result of the opportunistic nature of evolution. If an ancestral IGFBP was originally involved mainly in conditionally regulating the availability of IGFs to their receptors, then the context-specific inducible expression and secretion of IGFBP-5 would have presented a cue that could easily be coopted by evolution in order to trigger other adaptive responses to those same conditions. It is also worth considering that the one IGFBP gene present in the genome of amphioxus contains a nuclear localization sequence and transactivation activity, possibly indicating an ancestral role for IGF-independent functions (117). Further studies are needed in order to determine the circumstances in which this activity may play a role *in vivo*.

The paradox of IGFBPs in general, and IGFBP-5 in particular, is that they each seem to have many unique and important roles, and yet, loss of function experiments in model organisms have generally found either no phenotype or very minimal phenotypes when IGFBPs are deleted (11). This is especially puzzling for IGFBP-5 because it is the most evolutionarily conserved among all of the IGFBPs and yet IGFBP-5 knockout mice had normal growth, organ weights and body composition, and the only reported phenotype was a delay in mammary gland involution (67). But despite the apparent dispensability of IGFBP-5, no vertebrate species is known to have lost this gene. The emerging explanation for this apparent paradox is that IGFBP-5 acts mainly as conditional modulator of IGF signaling which confers an evolutionary advantage by facilitating the rapid adaptation of cell population growth rates to the needs of the environment. It is possible that we are not aware of all of the specific cases in which IGFBP-5 may conditionally act in different species. It is expected that these cases would not arise in laboratory conditions but would be much more likely to occur in response to the vicissitudes of life in the natural environment. The requirement of zebrafish IGFBP-5a for survival under low [Ca^2+^] stress (88) provides a paradigmatic example of the kind of circumstances in which previously undiscovered IGFBP-5 functions may be found. Future studies will clarify whether there are in fact other sets of conditions in which IGFBP-5 activity is required for survival.

## Author Contributions

CD conceived this review. CD and JA wrote this review.

### Conflict of Interest

The authors declare that the research was conducted in the absence of any commercial or financial relationships that could be construed as a potential conflict of interest.
