# Corrigendum: Insulin-Like Growth Factor Binding Protein-5 in Physiology and Disease

**DOI:** 10.3389/fendo.2020.00597

**Published:** 2020-08-28

**Authors:** Cunming Duan, John B. Allard

**Affiliations:** Department of Molecular, Cellular, and Developmental Biology, University of Michigan, Ann Arbor, MI, United States

**Keywords:** IGF signaling, AKT, mTOR, PAPP-A, STC, IGF-dependent, IGF-independent action

An author name was incorrectly spelled as **Cumming Duan**. The correct spelling is **Cunming Duan**.

The authors apologize for this error and state that this does not change the scientific conclusions of the article in any way. The original article has been updated.

